# Variation in North American bumble bee nest success and colony sizes under captive rearing conditions

**DOI:** 10.1093/jisesa/iead032

**Published:** 2023-06-05

**Authors:** James P Strange, Amber D Tripodi, Thuy-Tien T Lindsay, James D Herndon, Joyce Knoblett, Morgan E Christman, N Pinar Barkan, Jonathan B U Koch

**Affiliations:** Department of Entomology, The Ohio State University, Columbus, OH 43214, USA; United States Department of Agriculture, Agricultural Research Service, Pollinating Insect-Biology, Management, Systematics Research Unit, Logan, UT 84322, USA; United States Department of Agriculture, Agricultural Research Service, Pollinating Insect-Biology, Management, Systematics Research Unit, Logan, UT 84322, USA; Raleigh, NC 27604, USA; United States Department of Agriculture, Agricultural Research Service, Pollinating Insect-Biology, Management, Systematics Research Unit, Logan, UT 84322, USA; Department of Biology, Utah State University, Logan, UT 84322, USA; United States Department of Agriculture, Agricultural Research Service, Pollinating Insect-Biology, Management, Systematics Research Unit, Logan, UT 84322, USA; Department of Biology, Utah State University, Logan, UT 84322, USA; United States Department of Agriculture, Agricultural Research Service, Pollinating Insect-Biology, Management, Systematics Research Unit, Logan, UT 84322, USA; Department of Entomology, The Ohio State University, Columbus, OH 43214, USA; Department of Biology, Utah State University, Logan, UT 84322, USA; Department of Entomology, The Ohio State University, Columbus, OH 43214, USA; United States Department of Agriculture, Agricultural Research Service, Pollinating Insect-Biology, Management, Systematics Research Unit, Logan, UT 84322, USA

**Keywords:** *Bombus*, nest initiation, nesting success, commercialization, conservation

## Abstract

Of the 265 known bumble bee (*Bombus*) species, knowledge of colony lifecycle is derived from relatively few species. As interest in *Bombus* commercialization and conservation grows, it is becoming increasingly important to understand colony growth dynamics across a variety of species since variation exists in nest success, colony growth, and reproductive output. In this study, we reported successful nest initiation and establishment rates of colonies and generated a timeline of colony development for 15 western North American *Bombus* species, which were captively reared from wild-caught gynes from 2009 to 2019. Additionally, we assessed variation in colony size among 5 western North American *Bombus* species from 2015 to 2018. Nest initiation and establishment rates varied greatly among species, ranging from 5–76.1% and 0–54.6%, respectively. *Bombus griseocollis* had the highest rates of nest success across the 11-yr period, followed by *B. occidentalis*, *B. vosnesenskii*, and *B. huntii.* Furthermore, days to nest initiation and days to nest establishment varied among species, ranging from 8.4 to 27.7 days and 32.7 to 47 days. Colony size also differed significantly among species with *B. huntii* and *B. vosnesenskii* producing more worker/drone cells than *B. griseocollis*, *B. occidentalis*, and *B. vancouverensis.* Additionally, gyne production differed significantly among species with *B. huntii* colonies producing more gynes than *B. vosnesenskii.* Results from this study increase knowledge of systematic nesting biology for numerous western North American *Bombus* species under captive rearing conditions, which can further improve rearing techniques available to conservationists and researchers.

## Introduction

Bumble bees (Hymenoptera: Apidae: *Bombus*) are abundant and diverse pollinators with approximately 265 species primarily distributed throughout temperate, alpine, and subarctic ecosystems worldwide (Kremen et al. 2002, Berenbaum et al. 2007, Klein et al. 2007, Goulson 2010, Strange and Tripodi 2019, Williams and Jepsen 2020, Maebe et al. 2021). Bumble bees are primitively eusocial insects with overlapping generations within a colony, reproductive division of labor, and cooperative care of offspring. With the exception of some tropical species (Taylor and Cameron 2003), most bumble bee species have an annual life cycle that begins with the emergence of a mated gyne (reproductive female) from winter dormancy. The gyne searches for a suitable nesting site in abandoned rodent burrows, open grass tussocks, hollow logs, or above-ground man-made structures, and then forages for nest provisions (i.e., pollen and nectar) (Williams et al. 2014). The foundress gyne (now queen) constructs a wax honeypot for nectar storage within the nest, oviposits the first brood clutch on a pollen mass moistened with nectar, incubates the first clutch of brood, and continues to forage to provide food for the larvae (Williams et al. 2014). Each individual brood cell contains a single bumble bee larva. After the emergence of workers (female offspring) from brood cells, the queen ceases foraging. The queen then focuses efforts on oviposition, brood care, and maintaining social order, while workers perform tasks related to foraging, brood care (e.g., feeding developing larvae), colony maintenance (e.g., thermoregulation, cleaning), and defense. As floral resources become more abundant, the queen can produce offspring quickly, often resulting in rapid colony growth and development. Once the colony population peaks, usually in midsummer, the queen produces new reproductive gynes and drones (males). The new gynes and drones then leave the colony to feed and mate with offspring from other colonies. The foundress queen, workers, and drones then die; the newly mated gynes find a subterranean space and create a hibernaculum to undergo winter diapause, and the cycle continues (Alford 1975, Goulson 2010, Strange 2010, Williams et al. 2014, Koch et al. 2021).

Among the diverse *Bombus* species, there is considerable variation in colony establishment, growth, and development (Plowright and Jay 1966; Alford 1970; Pomeroy and Plowright 1980, Strange 2010). For example, nest site selection varies among species. While most species nest in subterranean habitats, such as abandoned rodent burrows, some others nest on soil surfaces in grass tussocks (Hobbs 1965a, 1965b, 1967, Macfarlane et al. 1994, Taylor and Cameron 2003) or in tree cavities, bird houses, or other elevated cavities (Macfarlane et al. 1994, Koch and Cane 2022). Furthermore, timing of gyne emergence from diapause is known to vary among species. Some *Bombus* emerge early in the spring, while others are not seen until after the emergence of early spring workers (Colla et al. 2011; Koch et al. 2012; Williams et al. 2014). These differences likely have evolutionary significance and may serve to reduce interspecific competition for nest sites and/or ephemeral spring floral resources, or to avoid social parasitism. In addition to species-specific nest sites and emergence timing, there is variation in colony size, nest success, and reproductive output among species. This variation can also be seen in laboratory-reared colonies, which is important to the utility of *Bombus* species as commercial pollinators.

Given these biological and evolutionary differences in colony-level factors, it is necessary to determine differences in nest initiation and establishment success, number of offspring and reproductives produced per colony, and colony development timelines among *Bombus* species (Macfarlane et al. 1994, Kwon et al. 2006, Yoneda 2008, Strange 2010). However, capturing this information in wild colonies is difficult given that the nests are cryptic and often below ground. Obtaining data from colonies reared from wild-caught queens in controlled laboratory environments can increase knowledge on *Bombus* species nesting biology, although rearing bumble bees for research purposes has resulted in varying degrees of success (Plowright and Jay 1966, Kearns and Thomson 2001, Evans et al. 2007, Salvarrey et al. 2013, Ptáček et al. 2015, Strange 2015, Carnell et al. 2020, Christman et al. 2022).

Recent efforts toward rearing bumble bees in captivity have improved techniques available to conservationists and researchers, which can be used to assess the commercial viability of nondomesticated *Bombus* species and to establish conservation techniques for imperiled species (Macfarlane et al. 1994, Strange et al. 2011, Christman et al. 2022, Rowe et al. 2023). For example, conservation propagation methods (i.e., assisted reintroductions) have been proposed as a recovery action to augment wild populations (Smith et al. 2020). In this study, we reported nest initiation success and colony establishment rates and generated a timeline of colony development for 15 western North American *Bombus* species, which were captively reared from wild-caught gynes from 2009 to 2019. Additionally, we assessed variation in colony size among 5 western North American *Bombus* species from 2015 to 2018. Results from this study increase knowledge of systematic nesting biology under captive-rearing conditions, which can further improve rearing techniques available to conservationists and researchers.

## Methods

### *Bombus* Rearing

A total of 3,355 gynes from 19 *Bombus* species were net-collected while foraging after emerging from winter dormancy in the western United States from 2009 to 2019. The captured gynes were transferred from nets to individual 7 × 3 cm plastic vials (W. W. Grainger Inc., Lake Forest, IL) modified to have ventilation holes (Rowe et al. 2023). The gynes were then transported in chilled, insulated containers to the United States Department of Agriculture–Agricultural Research Service, Pollinating Insect–Biology, Management, and Systematics Research Unit in Logan, UT. Once at the laboratory, colonies were initiated following methodology outlined in Rowe et al. (2023). Briefly, the gynes were moved into 15 cm × 15 cm × 10 cm plastic rearing chambers (Biobest Canada, Leamington, ON, Canada) in a designated rearing space maintained at 28 ± 2 °C and 65 ± 2% relative humidity in complete darkness. In approximately 14% of cases, gynes were paired with conspecifics to increase oviposition and nesting success, which is known as cofounding or pleometrosis (Sladen 1912, Plowright and Jay 1966, Bernasconi and Keller 1996, Ptáček et al. 2000, Strange 2010). Cofounding increases social stress, which generally results in the dominant gyne attacking and killing the other gyne. This then increases oviposition and establishment success rates (Sladen 1912, Plowright and Jay 1966, Bernasconi and Keller 1996, Ptáček et al. 2000, Strange 2010). After placement into rearing chambers, each gyne or pair of gynes was provisioned with 2 g of a multifloral honey bee collected pollen diet mixture and a feeder filled with 50% sugar solution with additives (artificial nectar) (Christman et al. 2022, Rowe et al. 2023). Preparation of multifloral pollen provisions and artificial nectar followed methodology described in Rowe et al. (2023). Following the production of brood, each colony was fed pollen and artificial nectar ad libitum. Once 5 workers eclosed, the nest was transferred to a 29 cm × 22 cm × 13 cm plastic colony box (Biobest Canada, Leamington, ON, Canada) to provide additional space for colony growth and development. Activities that involved colony handling, such as transfer, feeding, maintenance, and data recording, were conducted under red light to reduce disturbance to the colony.

### *Bombus* Nest Success

Nineteen *Bombus* species were reared over the 11-yr period at varying success rates to provide experimental colonies for scientific studies (Supplementary Table 1). However, 4 of the 19 species were collected at low rates and did not establish brood (*B. caliginosus* Frison = 1; *B. morrisoni* Cresson = 8; *B. pensylvanicus sonorus* De Geer = 1; *B. sitkensis* Nylander = 5); therefore, they were not included in our analysis. Colonies were checked at least every 3 days over the course of their development. Gynes were given approximately 21 days to produce brood after which time they were considered successful. Gynes that did not produce brood within this time range were culled. For each successful colony, the number of days to first brood and the number of days to first worker eclosion were recorded to determine timing of nest initiation and establishment (Strange 2010) and to generate a timeline of colony development in a controlled laboratory environment. Nest initiation was defined as evidence of the queen to produce brood, and nest establishment was defined as the ability of a queen to rear one adult female offspring (worker) from brood (Strange 2010). The timeline of colony development was documented as the average number of days to first observed brood (nest initiation) from nest installment and the average number of days to first observed worker (nest establishment) from nest installment for each *Bombus* species. The timeline indicates overall colony averages for days to nest initiation and establishment, not individual larval development.

### *Bombus* Colony Size

Given that these colonies were reared to provide colonies for other scientific studies, there is variation in data collection and documentation. From 2015 to 2018, 233 colonies across 9 species were reared in the controlled laboratory setting for the entirety of their lifecycle. Species were only included when more than 20 colonies were represented within the dataset; therefore, 4 species were excluded from our analysis (*B. appositus* Cresson = 1; *B. californicus* Smith = 1; *B. centralis* Cresson = 2; *B. melanopygus* Nylander = 1). Once the colony stopped producing brood, indicating the end of the colony’s lifecycle, the colony was dissected to assess variation in colony size among the captive-reared *Bombus* species. The total number of emerged workers/drones brood cells and emerged gyne brood cells was recorded for each colony. Worker and drone brood cells could not be differentiated due to similarities in cell sizes, so they were documented together. Meanwhile, gyne brood cells are produced toward the end of the colony lifecycle, are approximately twice the size of worker/drone brood cells (e.g., Koch and Cane 2022), and often occur on the top layer of the nest in clumps, which allow the gyne cells to be differentiated from worker/drone cells.

### Data Analysis

Two-sample *z*-tests for proportions were conducted for both nest initiation and nest establishment to compare success rates between colonies reared from individual or paired gynes. Individual analysis of variances (ANOVAs) were used to determine both differences in the total number of eclosed workers/drones and total number of eclosed gynes among species over the 4-yr period (*P* < 0.05). Tukey’s HSD post hoc tests were used when the ANOVAs produced significant results in order to determine which species means were significantly different. All conditions, including normality, variance, and independence, were met for the individual ANOVAs. Statistical analyses were conducted using base functions in R version 4.0.3 (R Core Team 2021).

## Results

### *Bombus* Nest Success

**Fig. 1.**
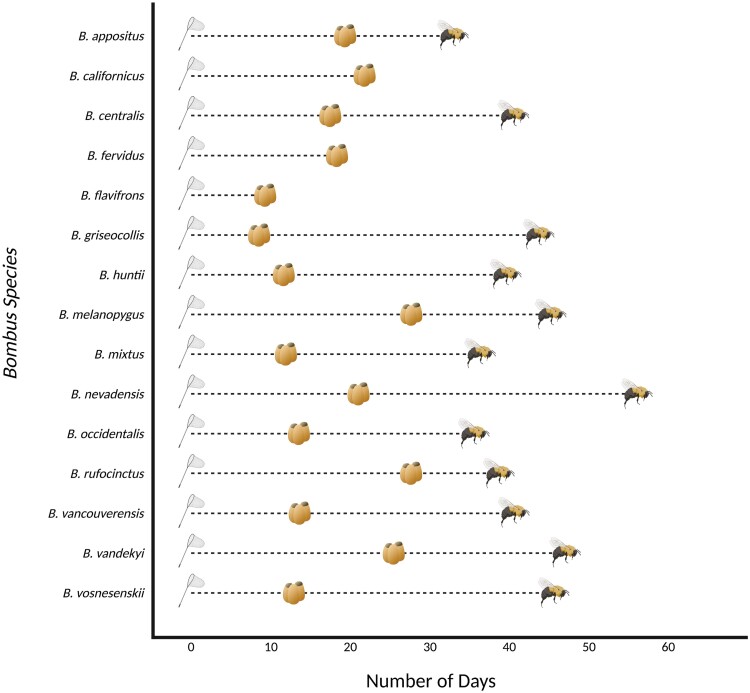
Timeline of colony development for western North American *Bombus* species. The gyne collection/installation day indicates the timeline’s starting point, which is represented by the aerial net. Average number of days to first observed brood (nest initiation) from nest installment for each *Bombus* species indicates the second point in the timeline, which is represented by the brood cells. Average number of days to first observed worker (nest establishment) from nest installment for each *Bombus* species indicates the final point in the timeline, represented by the bumble bee. The timeline indicates overall colony averages for days to nest initiation and nest establishment. Therefore, time between nest initiation and nest establishment is not equal to individual larval development.

From 2009 to 2019, 41.3% of gynes from 15 species produced brood cells (our criterion for nest initiation) (Table 1) and 18.8% of colonies from 12 species produced at least 1 worker (our criterion for nest establishment) in a controlled laboratory environment. Nest initiation and establishment rates varied greatly among species, ranging from 5% to 76.1% and 0% to 54.6%, respectively. *Bombus rufocinctus* Cresson (initiation: 5.2%; establishment: 3.4%) and *B. nevadensis* Cresson (initiation: 10%; establishment: 5%) had the lowest rates of nest success from 2009 to 2019. Meanwhile, *Bombus griseocollis* De Geer had the highest rates of nest success (initiation: 76.1%; establishment: 54.6%) across the 11-yr period, followed by *B. occidentalis* Greene (initiation: 59.2%; establishment: 34.8%), *B. vosnesenskii* Radoszkowski (initiation: 48.2%; establishment: 25.2%), and *B. huntii* Greene (initiation: 38.8%; establishment: 14.3%). Furthermore, days to nest initiation and days to nest establishment varied among species, ranging from 8.4 to 27.7 days and 32.7 to 47 days, respectively (Fig. 1).

**Table 1.** Rearing success of western North American *Bombus* species as defined by the production of brood (nest initiation) and emergence of a worker (nest establishment) from 2009 to 2019. Colony development of *Bombus* species within captivity as defined by days to nest initiation ± SD and days to nest establishment ± SD

*Bombus* species	Subgenus	Years produced	Successful nest initiation	Successful nest establishment	Days to first brood	Days to first worker
*B. appositus*	*Subterraneobombus*	2011, 2013, 2015, 2016, 2017, 2019	8/59 (13.6%)	4/59 (6.8%)	19.5 ± 10.7	32.7 ± 12.6
*B. californicus*	*Thoracobombus*	2014, 2015, 2016	2/8 (25%)	0/8 (0%)	21.5 ± 20.5	NA
*B. centralis*	*Pyrobombus*	2009, 2010, 2011, 2014, 2015, 2016, 2019	9/61 (14.8%)	2/61 (3.3%)	17.1 ± 5.5	40.0 ± 1.4
*B. fervidus*	*Thoracobombus*	2015, 2016, 2017	2/9 (22.2%)	0/9 (0%)	18.5 ± 3.5	NA
*B. flavifrons*	*Pyrobombus*	2013, 2014, 2015, 2016	1/8 (12.5%)	0/8 (0%)	9.0 ± 0.0	NA
*B. griseocollis*	*Cullumanobombus*	2011, 2015, 2016, 2017, 2018, 2019	156/205 (76.1%)	112/205 (54.6%)	8.4 ± 6.7	43.2 ± 13.4
*B. huntii*	*Pyrobombus*	2009, 2010, 2011, 2012, 2013, 2014, 2015, 2016, 2017, 2018, 2019	385/991 (38.8%)	142/991 (14.3%)	11.5 ± 9.9	39.2 ± 14.2
*B. melanopygus*	*Pyrobombus*	2011, 2014, 2015, 2016, 2018	25/140 (17.9%)	8/140 (5.7%)	27.4 ± 23.9	45.0 ± 13.4
*B. mixtus*	*Pyrobombus*	2012, 2015, 2016, 2017	5/14 (35.7%)	1/14 (7.1%)	12.0 ± 10.2	36.0 ± 0.0
*B. nevadensis*	*Bombias*	2011, 2013, 2015, 2017	2/20 (10%)	1/20 (5%)	21.5 ± 10.6	56.0 ± 0.0
*B. occidentalis*	*Bombus*	2009, 2010, 2011, 2012, 2014, 2015, 2016, 2017, 2018, 2019	119/201 (59.2%)	70/201 (34.8%)	13.7 ± 9.2	35.0 ± 7.1
*B. rufocinctus*	*Cullumanobombus*	2011, 2013, 2014, 2015, 2016, 2017, 2018, 2019	3/58 (5.2%)	2/58 (3.4%)	27.7 ± 19.9	38.0 ± 26.9
*B. vancouverensis*	*Pyrobombus*	2010, 2011, 2012, 2013, 2014, 2015, 2016, 2017, 2018, 2019	284/761 (37.3%)	86/761 (11.3%)	13.6 ± 9.8	40.2 ± 14.0
*B. vandykei*	*Pyrobombus*	2015, 2016	6/31 (19.4%)	4/31 (12.9%)	25.3 ± 15.9	47.0 ± 28.0
*B. vosnesenskii*	*Pyrobombus*	2014, 2015, 2016, 2017, 2018	373/774 (48.2%)	195/774 (25.2%)	12.9 ± 9.3	45.5 ± 14.9

Colonies reared from 2 gynes had significantly higher nest initiation (*z*-score = 5.59, df = 1, *P* < 0.001) rates per nest box compared with those reared from a single gyne. However, nest establishment rates did not differ between colonies reared from a single gyne or via cofounding (*z*-score = 1.05, df = 1, *p =* 0.15). Colonies reared from a single gyne had a nest initiation rate of 39.5% and a nest establishment rate of 18.5%. Meanwhile, colonies reared via cofounding had a nest initiation rate of 54.1% and a nest establishment rate of 20.8%.

### *Bombus* Colony Size

**Fig. 2.**
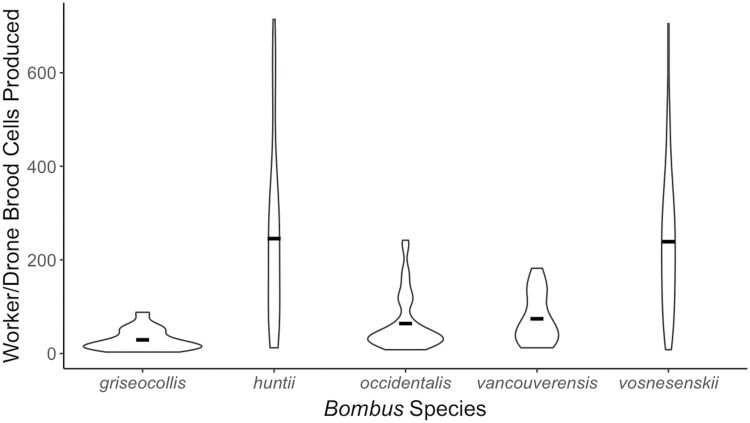
Distribution of worker/drone brood cells produced by *Bombus griseocollis*, *B. huntii*, *B. occidentalis*, *B. vancouverensis*, and *B. vosnesenskii* colonies from 2015 to 2018. Crossbars represent the mean.

**Fig. 3.**
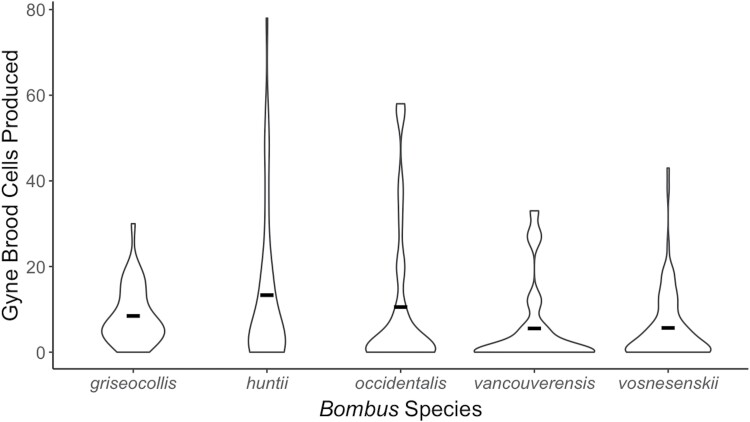
Distribution of gyne brood cells produced by *Bombus griseocollis*, *B. huntii*, *B. occidentalis*, *B. vancouverensis,* and *B. vosnesenskii* colonies from 2015 to 2018. Crossbars represent the mean.

Colony size parameters varied among species. Worker/drone brood cell production differed significantly among species (*F* = 28.79, df = 4, *P* < 0.001) (Fig. 2). Based on pairwise comparisons, *B. huntii* colonies were significantly larger than *B. griseocollis* (*P* < 0.001), *B. occidentalis* (*P* < 0.001), and *B. vancouverensis* Cresson (*P* < 0.001) colonies. Additionally, *B. vosnesenskii* colonies were significantly larger than *B. griseocollis* (*P* < 0.001), *B. occidentalis* (*P* < 0.001), and *B. vancouverensis* (*P* < 0.001) colonies. Gyne cell production also differed significantly among species (*F* = 3.358, df = 4, *P* = 0.011) (Fig. 3). Based on pairwise comparisons, *B. huntii* colonies produced significantly more gynes than *B. vosnesenskii* (*P* = 0.009).

## Discussion

This observational study provided a comprehensive documentation of the variation in rearing success rates, growth rates, and colony sizes among 15 western North American *Bombus* species, many of which have not been intensively studied. Furthermore, this study was the first to document rearing of 4 species in captivity, including *B. californicus*, *B. flavifrons* Eversmann, *B. mixtus* Cresson, and *B. vandykei* Frison, which adds to the literature available on nest success rates and colony development timelines. Overall, these results can be used to maximize captive-rearing success rates and enhance knowledge of systematic nesting biology and colony development in a controlled laboratory environment (Macfarlane et al. 1994, Kwon et al. 2006, Yoneda 2008, Strange 2010, Sarro et al. 2021).

Similar to previous studies, colonies reared via cofounding had significantly higher nest initiation rates per nest box compared to those reared from a single gyne, which supports that pairing gynes increase the probability that one individual will produce brood (Sladen 1912, Plowright and Jay 1966, Bernasconi and Keller 1996, Ptáček et al. 2000, Strange 2010). However, initiation and establishment rates with 2 gynes were not tested systematically across all species. Furthermore, high variability among species was observed in response to this method, which is consistent with Strange (2010). While cofounding can reduce labor, space, and resources needed for initiating colonies, this method should be tested for utility and efficacy in the target species prior to wide-scale implementation, particularly with species of conservation interest.

Across all species, *B. rufocinctus* and *B. nevadensis* had the lowest rates of nest success. Furthermore, several species (*B. californicus*, *B. fervidus* Fabricius, and *B. flavifrons*) did not establish a nest (rear a single worker to adulthood); however, this data may be biased due to low sample sizes. Meanwhile, *B. griseocollis* had the highest rate of nest success (quantified by nest initiation and establishment rates) across the 11-yr period, followed by *B. occidentalis*, *B. vosnesenskii*, and *B. huntii. Bombus griseocollis* initiated and established nesting at a rate of 76.1% and 54.6%, respectively, which was similar to findings from a study by Christman et al. (2022) (initiation: 70.6%; establishment: 52.8%). In addition to having the highest rate of nest success, *B. griseocollis* also had the fastest nest initiation rate among species, with an average of 8.4 ± 6.7 days to first brood. These results were also consistent with previous research from Christman et al. (2022), which documented an average of 7.6 ± 7 days to first brood for *B. griseocollis*. We were unable to compare nest establishment between our study and that of Christman et al. (2022) due to differences in the way in which days to nest establishment was calculated. Nest success or developmental timelines for *B. occidentalis*, *B. vosnesenskii*, and *B. huntii* have not been previously documented in the literature, although these species have been commercially produced. This information is likely not available given the proprietary nature of industry produced data.

Similar to our nest initiation and establishment results, colony size parameters varied among the 5 species. *Bombus huntii* and *B. vosnesenskii* had the largest colonies, producing an average of 245.5 ± 188.3 and 238.8 ± 151.6 worker/drone brood cells per colony, respectively. *Bombus huntii* also produced the most gynes, with an average of 13.3 ± 18.4 gyne cells per colony. These findings are of interest given that both of these species are now commercially available in North America, along with *B. impatiens* Cresson. The fact that they are within the subgenus *Pyrobombus*, have high rates of nest success, large colony sizes, and, in the case of *B. huntii*, sizeable gyne production may make them amenable for commercial production. *Pyrobombus* are pollen storers and have been reported to have lower rates of *Vairimorpha bombi* infection in wild populations (Sladen 1912; Velthuis and van Doorn 2006, Cordes et al. 2012, Malfi and Roulston 2014, Tripodi et al. 2014). Certain species of *Pyrobombus* have also been reported to emerge and establish nests early in the season, suggesting that these species have a longer time to persist and produce larger colonies compared with later emerging species (Hobbs 1967). Therefore, early nest establishment may increase colony nest success, making *Pyrobombus* species more amenable to mass production.

Given that these data were collected opportunistically as colonies were reared in a laboratory for experimental purposes, several biases are present. First, the 15 species were not reared consistently over the 11-yr period and varied in sample size. Over the 11-yr period in which these data were collected, minor changes in rearing protocols occurred to improve success rates, including alterations in bee diet preparation/composition. It is also important to note that the laboratory personnel responsible for rearing the colonies shifted over time. Additionally, given that these colonies were checked at least every 3 days, there is some degree of error associated with the documentation of the first observed brood and first observed worker. However, we assessed the interaction of year and species on nest initiation and establishment rates using generalized linear models and found that there was not a significant time or species effect on success rates. Additionally, the laboratory-reared colonies were used for a variety of studies. Colonies were often selected to be used in these studies based on their comparatively fast nest initiation/establishment rates and large colony sizes within species. Therefore, the reported results for the colony size parameters are likely underestimated. As such, results from this study are purely observational.

While these results are observational, this does not negate the importance of the findings reported in this study. On the contrary, these results enhance knowledge of systematic nesting biology and colony development in a controlled laboratory environment. This can provide insight into wild *Bombus* nesting biology and colony size, which can be difficult to study given that a majority of their life cycle is spent below ground. Furthermore, these rearing protocols could be employed on a variety of other bumble bees, including imperiled species, to help assist in their recovery. Rearing of ex situ colonies for assisted reintroductions has been identified as a last-resort conservation propagation method to mitigate the effects of population declines, particularly throughout the geographic range in which the species has been extirpated (López-Pujol et al. 2006, Shorthouse et al. 2012, Fritz and Chiari 2013, IUCN/SSC 2013; Draper et al. 2020). Therefore, captive rearing of bumble bees could prove to be a powerful tool in bumble bee conservation efforts.

## Supplementary Material

iead032_suppl_Supplementary_Material

## Data Availability

The data and code supporting the findings of this study are openly available on Zenodo at https://doi.org/10.5281/zenodo.7596923.

## References

[CIT0001] Alford DV. Bumblebees. London, UK: Davis-Poynter; 1975. p. 352.

[CIT0002] Berenbaum M , Bernhardt P, Buchmann S, Calderone N, Goldstein P, Inouye DW, Kevan P, Kremen C, Medellin RA, Ricketts T, et al. Status of pollinators in North America. Washington (DC): National Academies Press; 2007.

[CIT0003] Bernasconi G , Keller L. Reproductive conflicts in cooperative associations of fire ant queens (*Solenopsis invicta*). Proc R Soc B Biol Sci. 1996:263:509–513.

[CIT0004] Carnell JD , Page S, Goulson D, Hughes WOH. Trialing techniques for rearing long-tongued bumblebees under laboratory conditions. Apidologie. 2020:51:254266.

[CIT0005] Christman ME , Spears LR, Koch JBU, Lindsay TTT, Strange JP, Barnes CL, Ramirez RA. Captive rearing success and critical thermal maxima of *Bombus griseocollis* (Hymenoptera: Apidae): a candidate for commercialization? J Insect Sci. 2022:22:1–8. 10.1093/jisesa/ieac064 PMC9673274 36398850

[CIT0006] Cordes N , Huang WF, Strange JP, Cameron SA, Griswold TL, Lozier JD, Solter LF. Interspecific geographic distribution and variation of the pathogens *Nosema bombi* and *Crithidia* species in United States bumble bee populations. J Invertebr Pathol. 2012:109(2):209–216. 10.1016/j.jip.2011.11.005 22119631

[CIT0007] Draper FC , Baker TR, Baraloto C, Chave J, Costa F, Martin RE, Pennington RT, Vicentini A, Asner GP. Quantifying tropical plant diversity requires an integrated technological approach. Trends Ecol Evol. 2020:35(12):1100–1109. 10.1016/j.tree.2020.08.003 32912632

[CIT0008] Evans EC , Burns I, Spivak, M. Befriending bumble bees. Minneapolis (MN): University of Minnesota Extension; 2007.

[CIT0009] Fritz U , Chiari Y. Conservation actions for European pond turtles—a summary of current efforts in distinct European countries. Herpetol Notes. 2013:6:105.

[CIT0010] Goulson, D. Bumblebees: behaviour, ecology, and conservation. Oxford (UK): Oxford University Press; 2010.

[CIT0011] Hobbs GA. Ecology of species of *Bombus* Latr. (Hymenoptera: Apidae) in Southern Alberta. II. Subgenus *Bombias Robt*. Can Entomol. 1965a:97(2):120–128. 10.4039/ent97120-2

[CIT0012] Hobbs GA. Ecology of species of *Bombus* Latr. (Hymenoptera: Apidae) in Southern Alberta. III. Subgenus *Cullumanobombus* Vogt. Can Entomol. 1965b:97(12):1293–1302. 10.4039/ent971293-12

[CIT0013] Hobbs GA. Ecology of species of *Bombus* (Hymenoptera: Apidae) in Southern Alberta: VI. Subgenus *Pyrobombus*. Can Entomol. 1967:99(12):1271–1292. 10.4039/ent991271-12

[CIT0014] IUCN/SSC. Guidelines for reintroductions and other conservation translocations. Version 1.0. Gland (Switzerland): IUCN Species Survival Commission; 2013. p. viiii + 57.

[CIT0015] Kearns CA. , Thomson JD. Natural history of bumblebees. Denver (CO): University Press of Colorado; 2001.

[CIT0016] Klein A-M , Vaissiere BE, Cane JH, Steffan-Dewenter I, Cunningham SA, Kremen C, Tscharntke T. Importance of pollinators in changing landscapes for world crops. Proc R Soc B Biol Sci. 2007:274:303–313. 10.1098/rspb.2006.3721 PMC1702377 17164193

[CIT0017] Koch JBU , Cane JH. Pollen columns and a wax canopy in a first nest description of *Bombus* (*Cullumanobombus*) *morrisoni* (Apidae). Apidologie. 2022:53:1–9. 10.1007/s13592-022-00943-4

[CIT0018] Koch JBU , McCabe LM, Love BG, Cox-Foster D. Genetic and usurpation data support high incidence of bumble bee nest invasion by socially parasitic bumble bee, *Bombus insularis*. J Insect Sci. 2021:21:1–7. 10.1093/jisesa/ieab063 PMC8415179 34477874

[CIT0019] Kremen C , Williams NM, Thorp RW. Crop pollination from native bees at risk from agricultural intensification. Proc Natl Acad Sci USA. 2002:99(26):16812–16816. 10.1073/pnas.262413599 12486221 PMC139226

[CIT0020] Kwon YJ , Than KK, Suh SJ. New method to stimulate the onset of *Bombus terrestris* (Hymenoptera: Apidae) rearing: using worker helpers in the presence of frozen pupae. Entomol Res. 2006:36(4):202–207. 10.1111/j.1748-5967.2006.00041.x

[CIT0021] López-Pujol J , Zhang FM, Ge S. Plant biodiversity in China: richly varied, endangered, and in need of conservation. Biodivers Conser. 2006:15:3983–4026. 10.1007/s10531-005-3015-2

[CIT0022] Macfarlane RP , Patten KD, Royce LA, Wyatt BKW, Mayer DF. Management potential of sixteen North American bumble bee species. Melanderia. 1994:50:1–12.

[CIT0023] Maebe K , Hart AF, Marshall L, Vandamme P, Vereecken NJ, Michez D, Smagghe G. Bumblebee resilience to climate change, through plastic and adaptive responses. Glob Change Biol. 2021:27:4223–4237. 10.1111/gcb.15751 PMC13420811 34118096

[CIT0024] Malfi RL , Roulston TH. Patterns of parasite infection in bumble bees (*Bombus* spp.) of Northern Virginia. Ecol Entomol. 2014:39:17–29. 10.1111/een.12069

[CIT0025] Plowright RC , Jay SC. Rearing bumble bee colonies in captivity. J Apic Res. 1966:5:155–165. 10.1080/00218839.1966.11100149

[CIT0026] Pomeroy N , Plowright RC. Maintenance of bumble bee colonies in observation hives (Hymenoptera: Apidae). Can Entomol. 1980:112(3):321–326. 10.4039/ent112321-3

[CIT0027] Ptáček V , Borovec R, Pernova E. The two-queen cascade method as an alternative technique for starting bumble bee (*Bombus*, Hymenoptera: Apidae) colonies in laboratory conditions: a preliminary study. Psczelnicze Zaexyty Naukowe. 2000:44:305–309.

[CIT0028] Ptáček V , Votavová A, Komzáková O. Experience in rearing common carder bees (*Bombus pascuorum* Scop.), with some notes on three similar species: shrill carder bee (*B. sylvarum* L.), red-shanked carder bee (*B. ruderarius* Müll.), and brown-banded carder bee (*B. humilis* Ill.) (Hymenoptera: Apidae). Acta Univ Agric Silvic Mendelianae Brun. 2015:63:15351542.

[CIT0029] R Core Team. R: a language and environment for statistical computing. Vienna (Austria): R Foundation for Statistical Computing; 2021. https://www.R-project.org/.

[CIT0030] Rowe G , Hagadorn MA, Lindsay TTT, Malfi R, Williams NM, Strange JP. Chapter 20—production of bumblebees (Hymenoptera: Apidae) for pollination and research. In: Morales-Ramos JA, Guadalupe Rojas M, Shapiro-Ilan DI, editors. Mass production of beneficial organisms. 2nd ed. Cambridge, USA: Academic Press; 2023. p. 559–579. 10.1016/B978-0-12-822106-8.00004-X

[CIT0031] Salvarrey S , Arbulo N, Santos E, Invernizzi C. Artificial breeding of native bumblebees *Bombus atratus* y *Bombus bellicosus* (Hymenoptera, Apidae). Agrociencia (Montev). 2013:17:7582.

[CIT0032] Sarro E , Sun P, Mauck K, Rodriguez-Arellano D, Yamanaka N, Woodard SH. An organizing feature of bumble bee life history: worker emergence promotes queen reproduction and survival in young nests. Conserv Physiol. 2021:9:1–14. 10.1093/conphys/coab047 PMC8242224 34221405

[CIT0033] Shorthouse DJ , Iglesias D, Jeffress S, Lane S, Mills P, Woodbridge G, Sue M, Manning AD. The ‘making of’ the Mulligans Flat—Goorooyarroo experimental restoration project. Ecol Manag Restor. 2012:13:112–125. 10.1111/j.1442-8903.2012.00654.x

[CIT0034] Sladen FWL. The bumblebee, its life history and how to domesticate it. London (UK): Macmillan and Company; 1912.

[CIT0035] Smith TA , Strange JP, Evans EC, Sadd BM, Steiner JC, Mola JM, Traylor-Holzer K, editors. Rusty patched bumble bee, *Bombus affinis*, ex situ assessment and planning workshop: final report. Apple Valley (MN): IUCN SSC Conservation Planning Specialist Group; 2020. https://www.cpsg.org/content/rusty-patched-bumble-bee-ex-situ-assessment-and-planning-workshop-report.

[CIT0036] Strange JP. Nest initiation in three North American bumble bees (*Bombus*): gyne number and presence of honey bee workers influence establishment success and colony size. J Insect Sci. 2010:10(130):1–11. 10.1673/031.010.13001 20879924 PMC3016903

[CIT0037] Strange JP. *Bombus huntii*, *Bombus impatiens*, and *Bombus vosnesenskii* (Hymenoptera: Apidae) pollinate greenhouse-grown tomatoes in Western North America. J Econ Entomol. 2015:108(3):873–879. 10.1093/jee/tov078 26470206

[CIT0038] Strange JP , Koch JB, Gonzalez VH, Nemelka L, Griswold T. Global invasion by *Anthidium manicatum* (Linnaeus) (Hymenoptera: Megachilidae): assessing potential distribution in North America and beyond. Biol Invasions. 2011:13(9):2115–2133. 10.1007/s10530-011-0030-y

[CIT0039] Strange JP , Tripodi AD. Characterizing bumble bee (*Bombus*) communities in the United States and assessing a conservation monitoring method. Ecol Evol. 2019:9(3):1061–1069. 10.1002/ece3.4783 30805140 PMC6374645

[CIT0040] Taylor OM , Cameron SA. Nest construction and architecture of the Amazonian bumble bee (Hymenoptera: Apidae). Apidologie. 2003:34(4):321–331. 10.1051/apido:2003035

[CIT0041] Tripodi AD , Cibils-Stewart X, McCornack BP, Szalanski AL. *Nosema bombi* (Microsporidia: Nosematidae) and Trypanosomatid prevalence in spring bumble bee queens (Hymenoptera: Apidae: *Bombus*) in Kansas. J Kansas Entomol Soc. 2014:87(2):225–233. 10.2317/jkes130730.1

[CIT0042] Velthuis HHW , van Doorn A. A century of advances in bumblebee domestication and the economic and environmental aspects of its commercialization for pollination. Apidologie. 2006:37:421–451. 10.1051/apido:2006019

[CIT0043] Williams PH , Jepsen S. IUCN BBSG—bumblebee specialist group report 2019. Natural History Museum; 2020. p. 27. 10.13140/RG.2.2.15618.84166

[CIT0044] Williams P , Thorp R, Richardson L, Colla S. Bumble bees of North America: an identification guide. Princeton (NJ): Princeton University Press; 2014.

[CIT0045] Yoneda M. Induction of colony initiation by Japanese native bumble bees using cocoons of the exotic bumblebee *Bombus terrestris*. Entomol Sci. 2008:11(1):123–126. 10.1111/j.1479-8298.2007.00258.x

