# Supplementary material for: Variation in North American bumble bee nest success and colony sizes under captive rearing conditions
Source: J Insect Sci. 2023 Jun 5;23(3):10. doi: 10.1093/jisesa/iead032 (PMC10243899; doi:10.1093/jisesa/iead032)
Supplement: iead032_suppl_Supplementary_Material [file iead032_suppl_supplementary_material.docx]

**Supplementary Table 1**. Number of colonies reared per year per western North American *Bombus* species from 2009 to 2019. “–” indicates that no gynes were collected that year for that *Bombus* species.

| ***Bombus* Species** | 2009 | 2010 | 2011 | 2012 | 2013 | 2014 | 2015 | 2016 | 2017 | 2018 | 2019 |
| --- | --- | --- | --- | --- | --- | --- | --- | --- | --- | --- | --- |
| *B. appositus* | – | – | 19 | – | 7 | – | 5 | 8 | 19 | – | 1 |
| *B. californicus* | – | – | – | – | – | 1 | 6 | 1 | – | – | – |
| *B. caliginosis* | – | – | – | – | – | – | 1 | – | – | – | – |
| *B. centralis* | 3 | 1 | 1 | – | – | 1 | 33 | 20 | – | – | 2 |
| *B. fervidus* | – | – | – | – | – | – | 6 | 2 | 1 | – | – |
| *B. flavifrons* | – | – | – | – | 1 | 1 | 1 | 5 | – | – | – |
| *B. griseocollis* | – | – | 3 | – | – | – | 2 | 41 | 84 | 67 | 8 |
| *B. huntii* | 52 | 22 | 3 | 42 | 5 | 91 | 153 | 80 | 244 | 182 | 117 |
| *B. melanopygus* | – | – | 1 | – | – | 1 | 102 | 33 | – | 3 | – |
| *B. mixtus* | – | – | – | 1 | – | – | 6 | 6 | 1 | – | – |
| *B. morrisoni* | – | – | – | – | – | – | – | – | – | – | 8 |
| *B. nevadensis* | – | – | 1 | – | 9 | – | 9 | – | 1 | – | – |
| *B. occidentalis* | 3 | 3 | 2 | 10 | – | 37 | 7 | 61 | 14 | 30 | 34 |
| *B. pensylvanicus sonorus* | – | – | – | – | – | – | 1 | – | – | – | – |
| *B. rufocinctus* | – | – | 3 | – | 34 | 1 | 1 | 8 | 1 | 1 | 9 |
| *B. sitkensis* | – | – | – | – | – | – | 5 | – | – | – | – |
| *B. vancouverensis* | – | 9 | 19 | 65 | 2 | 86 | 160 | 146 | 92 | 95 | 87 |
| *B. vandykei* | – | – | – | – | – | – | 22 | 9 | – | – | – |
| *B. vosnesenskii* | – | – | – | – | – | 4 | 275 | 248 | 195 | 52 | – |
